# A randomized, double-blind, placebo-controlled clinical trial to evaluate pregabalin efficacy in the treatment of behavioral and psychological symptoms of dementia in patients with Alzheimer’s disease

**DOI:** 10.3389/fnins.2024.1460325

**Published:** 2024-11-27

**Authors:** Leyla Maleki, Fatemeh Mohammadian, Mahsa Panahishokouh, Niayesh Mohebbi

**Affiliations:** ^1^Department of Clinical Pharmacy, Faculty of Pharmacy, Tehran University of Medical Sciences, Tehran, Iran; ^2^Department of Psychiatry, Roozbeh Hospital, Tehran University of Medical Sciences, Tehran, Iran; ^3^Department of Clinical Pharmacy, Faculty of Pharmacy, Isfahan University of Medical Sciences, Isfahan, Iran; ^4^Research Center for Rational Use of Drugs, Tehran University of Medical Sciences, Tehran, Iran

**Keywords:** pregabalin, dementia, Alzheimer’s disease, psychological symptoms, behavioral symptoms

## Abstract

**Background:**

Behavioral and Psychological Symptoms of Dementia (BPSD) are common during Alzheimer’s disease and cause severe problems for patients and their caregivers.

**Objectives:**

To assess the therapeutic efficacy of Pregabalin in comparison with a placebo in treating BPSD in patients with Alzheimer’s disease (AD) visiting the memory and cognition clinic of Roozbeh Psychiatric Hospital in Tehran, Iran.

**Methods:**

A 12-week double-blind, randomized comparison of Pregabalin and placebo treatments was conducted in 53 patients with DSM-V diagnosis of dementia of Alzheimer’s type. They were randomly assigned to receive Pregabalin (doses: 50 titrated up to 300 mg/day) or a placebo. Clinical response was evaluated using the Neuropsychiatric Inventory (NPI), and the Behavioral Pathology in AD Rating Scale (BEHAVE-AD) scores compared with baseline. Side effects were also recorded carefully.

**Results:**

Patients receiving Pregabalin had better outcomes in comparison with patients receiving a placebo regarding both NPI and BEHAVE-AD scores after 12 weeks (*p*-value = 0.009 for NPI and *p*-value = 0.003 for BEHAVE-AD). There was also a statistically significant decrease in the treatment group depression sub-score regarding NPI and BEHAVE-AD (respectively, *p*-value =0.000, 0.003). The caregiver burden sub-score of the NPI test was also lower in patients receiving pregabalin (*p*-value = 0.000). There was no statistically significant difference between the occurrence of adverse effects between the two groups (*p*-value = 1.00).

**Conclusion:**

Pregabalin at a dose of 300 mg/day was well tolerated and associated with reductions in the severity and frequency of behavioral symptoms in patients with AD. Pregabalin could be considered a favorable choice for treating BPSD in adults with mild to moderate stages of Alzheimer ‘s-type dementia, considering its befitting safety profile.

**Clinical trial registration:**

https://www.irct.ir/trial/52750, identifier IRCT20201201049553N1

## Introduction

Dementia is an umbrella term for several progressive diseases that affect cognitive and behavioral abilities and significantly interfere with everyday activities. The most common type of dementia is Alzheimer’s disease (AD), which accounts for 60–70% of cases (Patterson, 2018). Alzheimer’s disease currently affects 50 million people worldwide according to the World Health Organization (2017).

More than 90 percent of cases diagnosed with dementia experience neuropsychiatric symptoms in their behavior known as Behavioral and Psychological Symptoms of Dementia (BPSD). BPSD includes mood disorders (e.g., depression and anxiety), psychosis (e.g., delusions and hallucinations), behavioral agitation (e.g., aggression), and other symptoms (e.g., sleep disorders and eating disorders) (Patterson, 2018). The presence of BPSD is associated with increased care costs and impaired quality of life; therefore, it increases the overall burden of dementia on patients, caregivers, and society (Schneider et al., 2005). There are two main approaches to the management of BPSD: non-pharmacological and pharmacological; the latter is usually considered when the symptoms are severe and there is a risk of self-harm or harm to caregivers (Ballard et al., 2009).

Antipsychotics, antidepressants, and mood stabilizer drugs have been considered for controlling psychosis and behavioral agitation symptoms (Ballard et al., 2009). Among them, antipsychotics are the most commonly prescribed medications despite their frequent side effects (Pollock et al., 2007). Risperidone is the only drug approved for use for no longer than 6–12 weeks due to the increased risk of mortality (Schneider et al., 2005). Previous trials were conducted for the use of antidepressants, especially citalopram, for the treatment of BPSD, but due to associated risks (QT prolongation and late onset of action), it is not considered the best option (Pollock et al., 2007).

Trazodone, a serotonin antagonist and reuptake inhibitor, has shown benefits in controlling agitation and sleep disturbances in dementia patients, due to being sedative. Nevertheless, its use is often limited by side effects, such as orthostatic hypotension and sedation (Tariot et al., 2015).

Tiapride, a selective dopamine D2 receptor antagonist, is another drug used for controlling aggression and agitation in dementia patients. Studies have shown it is effective in reducing these symptoms with a quite favorable safety profile. However, like other antipsychotics, it carries a risk of adverse effects such as sedation and extrapyramidal symptoms, which limit its use (Benabarre et al., 2016).

A more recent development in the pharmacological management of agitation in Alzheimer’s disease (AD) is brexpiprazole, a serotonin–dopamine activity modulator. Brexpiprazole was approved by the FDA for treating agitation associated with Alzheimer ‘s-related dementia, representing a significant advancement due to its safer profile compared to other antipsychotics (Grossberg et al., 2023). Its efficacy in reducing agitation and psychotic symptoms, combined with a lower risk of sedation and extrapyramidal side effects, makes it a better option for managing BPSD than other medications (Yu et al., 2024).

Despite these advances, the use of antipsychotics in dementia treatment remains controversial due to the increased risk of premature death. This risk is usually higher with older antipsychotics such as haloperidol, which has been associated with the highest mortality rates among dementia patients. While risperidone is the only antipsychotic approved for short-term use in this population, it is also associated with a heightened risk of death, especially when used for longer periods. Therefore, the careful selection of pharmacological interventions is necessary in balancing efficacy with safety (Schneider et al., 2005).

There are also small trials conducted for other types of medications such as carbamazepine, prazosin, and diphenhydramine, but due to limited evidence and potential side effects, they are not being prescribed (Sink et al., 2005; Pollock et al., 2007; American Geriatrics Society, 1998). Therefore, choosing an appropriate treatment strategy in favor of the patient and caregiver is challenging in controlling BPSD (Sink et al., 2005).

Pregabalin is a ‘gabapentinoid’ and in NBN-2 terminology a ‘voltage-gated calcium channel blocker’ *γ*-aminobutyric acid (GABA) analog but exerts its action by binding to the α2-*δ* protein, an auxiliary subunit of presynaptic voltage-gated calcium channels. By reducing presynaptic calcium, the release of excitatory neurotransmitters such as glutamate and substance P also decreases. This reduction is hypothesized to reduce network excitability, potentially leading to anxiolytic, antiepileptic, and analgesic effects (Supasitthumrong et al., 2019). Pregabalin and its analog gabapentin have shown benefits for the improvement of BPSD in several studies. The clinical evidence for the efficacy of pregabalin in the treatment of BPSD is limited, but there are certain reasons why it might be effective in improving BPSD (Supasitthumrong et al., 2019). The anxiolytic and analgesic effects of pregabalin have made it a possible option for chronic pain control in these patients, which is a potential cause of BPSD in patients with dementia (Kim et al., 2008; Supasitthumrong et al., 2019). Considering the mentioned mechanisms of pregabalin in improving BPSD symptoms, this double-blind, placebo-controlled trial is designed to evaluate the efficacy of pregabalin in the treatment of BPSD in patients with AD.

## Materials and methods

This was a double-blinded, placebo-controlled trial from July 2021 to January 2022 undertaken on patients referred to the memory and cognition clinic of Roozbeh Hospital, a referral center for psychiatry and neurology disorders affiliated with Tehran University of Medical Sciences (TUMS). The study was registered in the Iranian Registry of Clinical Trials (No. IRCT20201201049553N1). Patients 50 years old and older who have been diagnosed with AD based on the Diagnostic and Statistical Manual of Mental Disorders (DSM-V) were recruited for the study. All patients had a score of 24 to 10 on the Diagnostic and Statistical Manual of Mental Disorders (MMSE) and exhibited behavioral and psychological symptoms (BPSD) associated with dementia according to the Neuropsychiatric Inventory (NPI) and the Behavioral Pathology in AD Rating Scale (BEHAVE-AD) tests. Exclusion criteria included Parkinson’s disease, Lewy-body dementia, frontotemporal dementia, Pick disease, major psychological disorders, Multiple Sclerosis (MS), and a history of seizures or head trauma. Before participating, all patients or their caregivers signed an informed consent document approved by their study site’s Institutional Review Board. Patients were randomly assigned, in a 1:1 ratio with the random block randomization method, to 12 weeks of double-blind, flexible-dose treatment with Pregabalin (100–300 mg/day in two divided doses) or placebo groups. The patients assigned to the pregabalin group began treatment with 50 mg twice daily, which was increased to 100 mg twice daily at the end of the first week. Those assigned to placebo received the same number of pills with the same characteristics except for the active pharmaceutical ingredient. Both pregabalin and placebo were made by Sobhan Pharmaceutical Co. Cholinesterase inhibitors, memantine, and other medicines, including anticholinergics (up to 6 mg/day benztropine-equivalents), mood stabilizers (such as sodium valproate), selective serotonin reuptake inhibitors (SSRIs), antipsychotics, and melatonin, were permitted during the trial, and their dosage did not change unless there was a necessary indication. Information on the usage of these medications was recorded at baseline and analyzed to assess whether there were significant differences between the pregabalin and placebo groups in terms of concomitant pharmacotherapy. Patient compliance was assessed during the visits using the pill count method. To assess BPSD symptoms, including psychosis, agitation, aggression, and depression improvement, the scores for these symptoms were recorded and analyzed separately from either the NPI or BEHAVE-AD. Secondary measures included determining a total score for caregiver distress scores (the sum of caregiver distress scores for the NPI items, for outpatients). Patients were assessed in 3 visits: visit 0 (randomization), visit 1 (week 4), and visit 2 (week 12). Safety was assessed from spontaneous reports of treatment-emergent adverse events using the MedDRA dictionary, and from vital signs and physical examination by the clinician or reports by patients or their caregivers. A special form for reporting adverse events was designed and filled out by the clinician during each visit.

NPI and BEHAVE-AD were employed to assess BPSD. NPI is the main test designed for the assessment of behavioral functioning in 12 sub-domains, which include delusions, hallucinations, agitation/aggression, dysphoria, anxiety, euphoria, apathy, disinhibition, irritability/lability, aberrant motor activity, night-time behavioral disturbances, as well as appetite and eating abnormalities, BEHAVE-AD is another prominent test to assess BPSD and examines the mentioned domains. The questions are asked by patients’ caregivers, and based on a scale designed to determine the severity and frequency of symptoms, the scores of each part of both tests are determined, and the final score is determined from the sum of the different subsets (Cummings, 1997; Reisberg et al., 1997).

### Statistical analysis

All patients with a baseline and at least one post-baseline score for the NPI Total were included in the last-observation-carried-forward (LOCF) analysis of mean change from baseline to endpoint. According to the results of the Kolmogorov–Smirnov test, all data have a normal distribution and parametric tests have been used. Continuous variables were analyzed using longitudinal analysis. To compare the basic characteristics of the studied patients at the beginning of the study, an independent sample t-test was used for quantitative variables, and qualitative variables were compared between two groups with Fisher’s exact test. In this study, all analyses were conducted SPSS^1^ for Windows version 26.0. A level of *p* < 0.05 was considered statistically significant.

## Results

**Figure 1:**
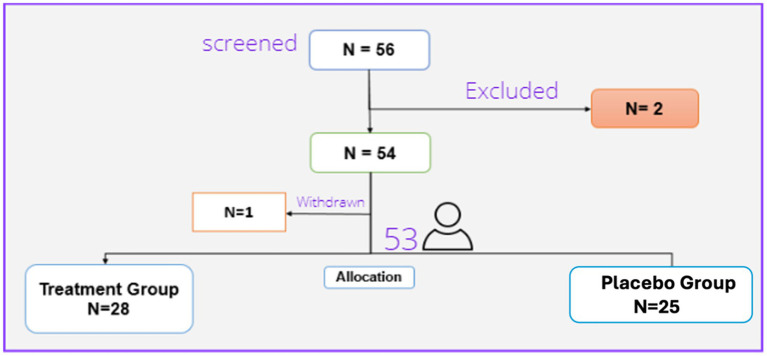
Patients’ flow diagram.

Overall, 56 patients were screened, of whom two were excluded due to meeting the exclusion criteria and refusing to sign the contest form, and 54 were randomized. One patient in the treatment group was withdrawn due to the withdrawal of consent. So, we had 28 patients in the treatment group and 25 patients in the placebo group (Figure 1).

Thus, 53 patients (29 females and 24 males) finally completed the trial. These were randomly assigned to the treatment (28 patients) or placebo (25 patients) groups. There were no statistically significant differences regarding the baseline characteristics between the two studied groups. Table 1 presents the demographic characteristics of the patients.

**Table 1:** Baseline demographic and characteristics of the studied patients.

Characteristics	Treatment group	Placebo group	*p* value
Gender (male/female) *N* (%)	12 (42.9%)/16 (57.1%)	12 (48%)/13 (52%)	0.52
Age (years), mean ± SD (range)	73.93 ± 9.68 (56–91)	74.5 ± 7.04 (63–91)	0.80
Patients’ education levels (years) mean ± SD	5.43 ± 7.21	8.88 ± 5.59	0.058

Like other baseline characteristics, the two treatment groups had no different NPI. Total (*p*-value =0.87), BEHAVE-AD (*p*-value =0.49), NPI.Psychosis (*p*-value =0.65), NPI.agitation (*p*-value =0.07), NPI.aggression (*p*-value =0.09), NPI.depression (*p*-value =0.06), NPI.caregiver (*p*-value =0.2) BEHAVE-AD.psychosis (*p*-value =0.9), BEHAVE-AD.agitation (*p*-value =0.06), BEHAVE-AD.aggression (*p*-value =0.88), and BEHAVE-AD.depression (*p*-value =0.3) score using the Independent Samples *T*-test at baseline.

Table 2 shows no significant differences between the two groups for all medications except for anti-psychotics. Due to the small sample size, the difference in outcomes was evaluated by a multivariate longitudinal analysis. Based on the results of the longitudinal model, the use of antipsychotics did not have any significance on the mean difference of total score of NPI, but had a significance in the mean difference of total score of behave-AD resulting in 8.91 higher score in patients taking anti-psychotics compared to patients not taking anti-psychotics. These findings somehow also show that the patients receiving anti-psychotics showed more behavioral symptoms, based on the same test, receiving Pregabalin versus Placebo had a significant difference in NPI and Behave-AD total scores, indicating that patients who received Pregabalin had lower scores in both tests (5.8 and 11.5 respectively) (Table 3), The results for week 4, week 12 (as the time factor) age and treatment group has been further discussed with details in the article.

**Table 2:** Concomitant pharmacotherapy analysis.

Medication	Placebo	Treatment	*p*-value
Memantine (%)	48	53	0.524
Antipsychotic (%)	44	82	0.0039
Melatonin (%)	75	50	0.085
Anti-depressant (%)	96	93	0.871
Cholinesterase inhibitors (%)	100	100	1.000
Anticonvulsant (%)	8.3	2.6	0.228

**Table 3:** Comparison of mean difference with longitudinal analysis.

	Contributing factor	Mean difference (Effect estimate)	*T*	Sig		Contributing factor	Mean difference	*T*	Sig
NPI total score	Intercept	39.07	3.87	0.000	BEHAVE-AD total score	Intercept	20.36	1.15	0.250
	Week 4	−6.86	−3.08	0.002		Week 4	−13.88	−3.55	0.500
	Week 12	−8.30	−3.72	0.000		Week 12	−18.94	−4.85	0.000
	Age	0.06	0.51	0.610		Age	0.16	0.80	0.423
	Sex	−1.80	−0.90	0.364		Sex	0.29	0.08	0.932
	Treatment group	−5.80	−2.63	0.009		Treatment group	−11.55	−2.99	0.003
NPI psychosis sub-score	Intercept	3.85	0.87	0.383	BEHAVE-AD psychosis sub-score	Intercept	11.77	1.03	0.301
	Week 4	−2.20	−2.22	0.025		Week 4	−7.54	−3.00	0.003
	Week 12	−3.66	−3.75	0.000		Week 12	−9.83	−3.91	0.000
	Age	0.06	1.19	0.232		Age	0.06	0.50	0.613
	Sex	0.37	0.43	0.666		Sex	−0.34	−0.15	0.876
	Treatment group	−1.50	−1.56	0.119		Treatment group	−2.71	−1.90	0.275
NPI agitation sub-score	Intercept	5.61	1.62	0.107	BEHAVE-AD agitation sub-score	Intercept	4.69	0.77	0.440
	Week 4	−0.83	−1.08	0.280		Week 4	−0.33	−0.25	0.800
	Week 12	−0.60	−0.87	0.432		Week 12	−0.28	−0.21	0.833
	Age	0.02	0.72	0.470		Age	0.00	−0.08	0.933
	Sex	0.29	0.08	0.932		Sex	−0.12	−0.12	0.898
	Treatment group	−11.55	−2.99	0.003		Treatment group	−2.11	−1.59	0.114
NPI aggression sub-score	Intercept	1.31	0.60	0.543	BEHAVE-AD aggression sub-score	Intercept	−1.42	−0.22	0.842
	Week 4	−0.50	−1.60	0.278		Week 4	−2.45	−1.73	0.085
	Week 12	−0.49	−1.02	0.305		Week 12	−3.07	−2.17	0.031
	Age	0.00	0.29	0.768		Age	−0.03	−0.45	0.648
	Sex	0.71	1.67	0.096		Sex	2.44	−0.45	0.054
	Treatment group	−0.38	−0.82	0.412		Treatment group	−1.64	−1.17	0.240
NPI depression sub-score	Intercept	5.98	3.50	0.000	BEHAVE-AD depression sub-score	Intercept	6.83	1.28	0.200
	Week 4	−0.66	−1.74	0.082		Week 4	−1.75	−1.49	0.137
	Week 12	−0.50	−1.34	0.179		Week 12	−2.52	−2.15	0.033
	Age	0.00	0.03	0.968		Age	0.00	−0.14	0.885
	Sex	−0.22	−0.65	0.512		Sex	0.91	0.87	0.383
	Treatment group	−1.39	−3.74	0.000		Treatment group	−3.40	−2.93	0.003

A paired *t*-test was done to assess the pre and post-treatment effects both in treatment and control groups. This analysis was followed by conducting a longitudinal multivariate analysis of the data to determine the effect of time and treatment on outcomes, specifically sub-scores of both the Behave-AD and NPI tests (Table 4).

**Table 4:** Analysis for paired data, at time 0 versus 12 weeks for treatment and control groups.

Measure	Group	Mean difference	*T* value	df	*p*-value
NPI score	Treatment	−9.78	−3.892	26	0.000
NPI score	Control	−6.13	−2.1092	22	0.046
Behave-Ad score	Treatment	−19.56	−4.8044	26	0.000
Behave-Ad score	Control	−18.43	−3.1311	22	0.004

Regarding the longitudinal analysis, patients receiving Pregabalin had better outcomes in comparison with patients receiving placebo regarding both NPI and BEHAVE-AD scores after 12 weeks (*p*-value = 0.009 for NPI and *p*-value = 0.003 for BEHAVE-AD). There was also a statistically significant decrease in the treatment group depression sub-score regarding NPI and BEHAVE-AD (respectively, *p*-value =0.000, 0.003) (Table 3). The caregiver burden sub-score was also assessed with NPI and was lower in patients receiving pregabalin (*p*-value = 0.000).

According to the results of Table 3, the overall scores of the BEHAVE-AD and NPI tests have changed over time in the whole population. It also shows that the patients in the treatment group have a lower score on both tests compared to the control group.

### Safety

In the 12 weeks of the trial, 16 adverse events were reported and assessed based on the WHO causality assessment system (World Health Organization, 2013). There was no incidence of serious adverse events, and no adverse events caused withdrawal. The highest grade of adverse event reported was grade 3 according to the CTCAE grading system (National Cancer Institute, 2017).

There were 10 adverse events, including constipation, dizziness, gait disturbance, headache, nausea, facial edema, and blurred vision, reported in the treatment group, and 6 adverse events, including dizziness, headache, peripheral neuropathy, and weight gain, in the control group. There was no statistically significant difference between the manifestations of adverse events between the two groups (*p*-value = 1.00).

## Discussion

AD is a neurodegenerative disease with progressive and severe dementia along with neuropsychiatric symptoms. Some common drug options are used to improve behavioral symptoms. Based on various studies, gabapentinoids have shown positive effects in the treatment of mental and behavioral symptoms of dementia (Supasitthumrong et al., 2019). Among them, pregabalin is safe and prescribed for the management of anxiety and pain in the elderly based on case reports and meta-analyses (Kim et al., 2008; Supasitthumrong et al., 2019). There are limited clinical studies regarding the effect of this drug on the improvement of patients’ neuropsychiatric symptoms of dementia. The present study is a clinical trial to evaluate the potential of pregabalin to improve BPSD in AD patients, which was developed on two parallel treatment groups characterized as randomized, double-blind, and placebo-controlled.

Our results demonstrated that patients with mild to moderate AD with BPSD receiving Pregabalin showed statistically significant improvement in BPSD after 3 months of treatment. The clinical evidence of these findings can be underlined by the improvements seen in the measurement of NPI and BEHAVE-AD scores in the pregabalin group compared to the placebo. The depression sub-score of these tests also decreased. The adverse events in the treatment group in this study were similar to those in the placebo group.

In connection with the interpretation of the above results, the details of the results are discussed and compared with similar studies. According to a study that assessed the results of 15 case studies on the effects of gabapentinoid drugs on the psychological and behavioral symptoms of dementia, most patients with Alzheimer’s disease or vascular dementia treated with at least 200–1,200 mg of pregabalin had a significant improvement in psychological symptoms. The researchers of this study believe that due to the extensive side effects of drugs such as carbamazepine, citalopram, and risperidone, gabapentinoid drugs can be a suitable option for the elderly with this disorder. Like our study, in this study as well, in terms of safety, there was no significant difference between the adverse events observed in the treatment and placebo groups (*p*-value = 1.00) (Supasitthumrong et al., 2019).

The results of a retrospective study regarding the effectiveness of pregabalin in improving the psychological and behavioral symptoms of patients with neurocognitive disorders (including Alzheimer’s) showed that pregabalin could be considered as an alternative to benzodiazepines for anxiety disorders in these patients. The limitations of this study include the small sample size and its retrospective nature (Novais et al., 2019). Nevertheless, considering the positive effect of pregabalin in reducing the anxiety symptoms of patients, the positive outcome of this study regarding the use of pregabalin can be considered consistent with the results of our study.

Various studies, including systematic reviews and case reports, have pointed to the potential benefits of gabapentin in the treatment of BPSD (Kim et al., 2008; Tampi et al., 2012; Cooney et al., 2013). Such as a clinical trial study conducted on AD patients with BPSD symptoms. The results consistent with our study suggest pregabalin as a potential treatment option for BPSD (Supasitthumrong et al., 2019).

Several studies have analyzed the factors affecting caregiver burden in dementia patients. The severity of BPSD is one of the most prominent factors in increasing the burden on caregivers. Based on a longitudinal analysis conducted on 720 dementia patients over 3 years, neuropsychiatric symptoms are one of the factors affecting caregivers’ burden (Connors et al., 2020). The results of our study showed that the burden score of NPI caregivers in the treatment group decreased along with the decrease of the NPI score in patients, which is consistent with the results of this study.

We found that pregabalin at a dose of 300 mg/day was well tolerated and associated with reduced severity and frequency of behavioral symptoms in subjects with dementia. Pregabalin could be considered a favorable choice for treating BPSD in adults with mild to moderate dementia of the AD type considering its befitting safety profile.

## Data Availability

The raw data supporting the conclusions of this article will be made available by the authors, without undue reservation.

## Footnotes

^1^Statistical package for the social sciences.

## References

[ref1] American Geriatrics Society (1998). The management of chronic pain in older persons: AGS panel on chronic pain in older persons. J. Am. Geriatr. Soc. 46, 635–651. doi: 10.1111/j.1532-5415.1998.tb01084.x 9588381

[ref2] Ballard C. G. Gauthier S. Cummings J. L. Brodaty H. Grossberg G. T. Robert P.. (2009). Management of agitation and aggression associated with Alzheimer disease. Nat. Rev. Neurol. 5, 245–255. doi: 10.1038/nrneurol.2009.39 19488082

[ref3] Benabarre A. Sierra P. Lobo A. Segura C. (2016). Tiapride in dementia: efficacy and safety profile. Hum. Psychopharmacol. Clin. Exp. 37, e2842–e2216. doi: 10.1002/hup.2842, PMID: 35313032

[ref5] Connors M. H. Seeher K. Teixeira-Pinto A. Woodward M. Ames D. Brodaty H. (2020). Dementia and caregiver burden: a three-year longitudinal study. Int. J. Geriatr. Psychiatry 35, 250–258. doi: 10.1002/gps.5244, PMID: 31821606

[ref1002] Cooney C. Murphy S. Tessema H. Freyne A. (2013). Use of low-dose gabapentin for aggressive behavior in vascular and Mixed Vascular/Alzheimer Dementia. J. Neuropsy. Clinical Neurosc. 25, 120–125. doi: 10.1176/appi.neuropsych.12020048, PMID: 23686029

[ref6] Cummings J. L. (1997). The neuropsychiatric inventory: assessing psychopathology in dementia patients. Neurology 48, 10S–16S. doi: 10.1212/WNL.48.5_Suppl_6.10S 9153155

[ref7] Grossberg G. T. Kivipelto M. Hampel H. (2023). Brexpiprazole in the treatment of agitation associated with Alzheimer’s disease: a review. Brain Sci. 13:397. doi: 10.3390/brainsci13030397, PMID: 36979208 PMC10046771

[ref8] Kim Y. Wilkins K. M. Tampi R. R. (2008). Use of gabapentin in the treatment of behavioural and psychological symptoms of dementia: a review of the evidence. Drugs Aging 25, 187–196. doi: 10.2165/00002512-200825030-00002, PMID: 18331071

[ref9] National Cancer Institute. (2017). Common terminology criteria for adverse events. Available at: https://ctep.cancer.gov/protocoldevelopment/electronic_applications/docs/ctcae_v5_quick_reference_5x7.pdf

[ref10] Novais T. Doutone A. Gombault C. Krolak-Salmon P. Lepetit A. Mouchoux C. (2019). Description of the treatment course by pregabalin for anxiety in patients with a major neurocognitive disorder. J. Clin. Psychopharmacol. 39, 261–263. doi: 10.1097/JCP.0000000000001029, PMID: 30939590

[ref11] Patterson C. (2018). World Alzheimer report 2018. The state of the art of dementia research: new frontiers. London: Alzheimer’s Disease International.

[ref12] Pollock B. G. Mulsant B. H. Rosen J. Sweet R. A. Mazumdar S. Blakesley R. E.. (2007). A double-blind comparison of citalopram and risperidone for the treatment of behavioral and psychotic symptoms associated with dementia. Am. J. Geriatr. Psychiatry 15, 942–952. doi: 10.1097/JGP.0b013e3180cc1ff5, PMID: 17846102

[ref13] Reisberg B. Auer S. R. Monteiro I. M. (1997). Behavioral pathology in Alzheimer's disease (BEHAVE-AD) rating scale. Int. Psychogeriatr. 8, 301–308. doi: 10.1017/S1041610297003529 9154579

[ref14] Schneider L. S. Dagerman K. S. Insel P. (2005). Risk of death with atypical antipsychotic drug treatment for dementia: meta-analysis of randomized placebo-controlled trials. JAMA 294, 1934–1943. doi: 10.1001/jama.294.15.1934 16234500

[ref15] Sink K. M. Holden K. F. Yaffe K. (2005). Pharmacological treatment of neuropsychiatric symptoms of dementia: a review of the evidence. JAMA 293, 596–608. doi: 10.1001/jama.293.5.596 15687315

[ref16] Supasitthumrong T. Bolea-Alamanac B. M. Asmer S. Woo V. L. Abdool P. S. Davies S. J. (2019). Gabapentin and pregabalin to treat aggressivity in dementia: a systematic review and illustrative case report. Br. J. Clin. Pharmacol. 85, 690–703. doi: 10.1111/bcp.13844, PMID: 30575088 PMC6422659

[ref17] Tampi R. R. Ozkan B. Williamson D. (2012). Gabapentin for the treatment of behavioral and psychological symptoms of dementia. Adv. Alzh. Dis. 1, 13–16. doi: 10.4236/aad.2012.12002

[ref18] Tariot P. N. Schneider L. S. Cummings J. L. (2015). Trazodone for managing behavioral disorders in dementia patients. Curr. Pharm. Des. 21, 3343–3351. doi: 10.2174/1381612821666150619092236, PMID: 26088119

[ref19] World Health Organization. (2013). The use of the WHO-UMC system for standardized case causality assessment. Available at: https://www.who.int/publications/m/item/WHO-causality-assessment.

[ref20] World Health Organization. (2017). Dementia. Available at: http://www.who.int/mediacentre/factsheets/fs362/en/.

[ref1003] Yu. (2024). Use of brexpiprazole in the treatment of agitation associated with dementia due to Alzheimer’s disease: post hoc efficacy data. Presen Alzheimer’s Assoc. Intern. Conference (AAIC). doi: 10.2165/00002512-202425030-00001

